# Supplementary material for: Individual and public health consequences associated with a missed diagnosis of pulmonary tuberculosis in the emergency department: A retrospective cohort study
Source: PLoS One. 2021 Mar 22;16(3):e0248493. doi: 10.1371/journal.pone.0248493 (PMC7984634; doi:10.1371/journal.pone.0248493)
Supplement: Attachment — Submitted filename: PlosONE - rebuttal.doc [file pone.0248493.s002.doc]

**Dr. Courtney Heffernan**

**TB Program Evaluation and Research Unit**

**Department of Medicine,** Division of Pulmonary Medicine

U N I V E R S I T Y O F

A L B E R T A

8334A Aberhart Centre One	Tel: 780.407.1427

11402 University Avenue, Edmonton, Alberta, Canada T6G 2J3	Fax: 780.407.1429

February 19, 2021

Dr.  Emily Chenette

Deputy Editor-in-Chief, *PLOS One*

Dear Dr. Chenette,

**RE: Manuscript** **PONE-D-21-00447** **titled, *“Individual and public health consequences associated with a missed diagnosis of pulmonary tuberculosis in the emergency department: A retrospective cohort study”***

We thank the editorial board, and the reviewers for providing us with feedback to make a resubmission of this work possible. We have responded to the issues raised by the Editorial board, and by the reviewers, as below.

1. Please ensure that your manuscript meets PLOS ONE's style requirements, including those for file naming.

**We have reformatted our manuscript and our files according to the guidelines provided.**

2. Please note that all PLOS journals ask authors to adhere to our policies for sharing of data and materials: <https://journals.plos.org/plosone/s/data-availability>. According to PLOS ONE’s Data Availability policy, we require that the minimal dataset underlying results reported in the submission must be made immediately and freely available at the time of publication. As such, please remove any instances of 'unpublished data' or 'data not shown' in your manuscript and replace these with either the relevant data (in the form of additional figures, tables or descriptive text, as appropriate), a citation to where the data can be found, or remove altogether any statements supported by data not presented in the manuscript.

**We have removed the statement supported by the text, “data not shown” as it was not directly relevant to our findings.**

Reviewer #1: Comments:
Study conducted by Heffernan C et al has been designed very nicely to find out the missed diagnosed TB patients at the level of emergency departments. This will create health hazardous and dangerous exposures to the health care workers as well as the house hold contacts, especially in pediatrics groups. In case of previously treated TB patients, this may developed drug-resistant TB among the common population.
I am happy to accept this article with single correction.

Method:
• L. no. 102 add reference.

**Thank you for the careful review – the text ‘ref’ was included in the text in error, so we have deleted this text rather than adding an additional reference.**

Reviewer #2: The specificity and sensitivity of smear test for diagnosis of TB.
Since it was a retrospective cohort study therefore the standard mode is acceptable. But the authors view on advances in diagnosis of TB such as gene expert.

**Thank you for this comment; we have added a sentence (see lines: 345-348) about the future potential of Gene Xpert in supporting the diagnosis of pulmonary TB.**

Sincerely,

Courtney Heffernan, PhD; Corresponding Author
